# Changes in the ocular surface microbiome of patients with coronavirus disease 2019 (COVID-19)

**DOI:** 10.3389/fmicb.2024.1389139

**Published:** 2024-07-08

**Authors:** Jia Lin, Jingrao Wang, Jiaoyang Feng, Rui Zhu, Yu Guo, Yueyan Dong, Hong Zhang, Xin Jin

**Affiliations:** Department of Ophthalmology, The First Affiliated Hospital of Harbin Medical University, Harbin, China

**Keywords:** COVID-19, COVID-19 recovery period, ocular surface microbiome, 16S rRNA amplicon sequencing, ocular surface

## Abstract

**Purpose:**

To elucidate the reasons behind the increased incidence of ocular disease in patients with coronavirus disease 2019 (COVID-19), this study delved deeper into the specific effects of COVID-19 on patients’ ocular surface microbiome (OSM) and investigated its relationship with the increased incidence of ocular disease.

**Methods:**

In this study, conjunctival sac swabs were collected from 43 participants for 16S rRNA amplicon sequencing. The participants were categorized into three groups based on their COVID-19 status: the control group (C group) consisted of 15 participants who showed no evidence of COVID-19, the experimental group (E group) included 15 participants who tested positive for COVID-19, and the COVID-19 recovery period group (R group) comprised 13 participants.

**Results:**

In the comparison of alpha diversity, group E had a higher Shannon, Chao1 and Goods coverage index. When comparing beta diversity, groups E and R were more similar to each other. At the phylum level, although the OSM of the three groups was dominated by *Proteobacteria*, *Actinobacteriota*, *Bacteroidota* and *Firmicutes*, the compositional proportions were significantly different. At the genus level, the dominant species in the three OSM groups were significantly different, with *Pseudomonas* becoming the dominant genus in groups E and R compared to group C, and the abundance of *Ralstonia* decreasing significantly.

**Conclusion:**

This study provides additional evidence supporting the association between the OSM and COVID-19, which contributes to our understanding of the potential mechanisms underlying ocular symptoms and complications associated with COVID-19 in the future.

## 1 Introduction

The worldwide outbreak of coronavirus disease 2019 (COVID-19), caused by severe acute respiratory syndrome coronavirus 2 (SARS-CoV-2), has led to a considerable number of confirmed cases and deaths. As of 6 September 2023, there have been more than 771 million confirmed cases of COVID-19, including more than 6.97 million deaths reported by the World Health Organization (2023). Although respiratory symptoms and myalgias are the primary clinical manifestations in patients with COVID-19, there is evidence that 2.26% of COVID-19 patients initially present with ocular symptoms, which include conjunctival congestion, dry eye, conjunctival secretion, tearing, and ocular pain (Aggarwal et al., 2020; Chen et al., 2020). A study conducted in Italy has shown that direct contact with airborne droplets of SARS-CoV-2 through the conjunctiva and subsequent spread throughout the body via the nasolacrimal duct may be a potential route of infection. This finding suggests that presence of SARS-CoV-2 infection on the ocular surface in patients with COVID-19 (Azzolini et al., 2021). With the lifting of the quarantine policy and the increase in outpatient visits, it is becoming increasingly clear that SARS-CoV-2 infection is linked to dry eye, conjunctivitis, keratitis, Steven-Johnson syndrome (SJS), and other conditions (Lima et al., 2021; Saldanha et al., 2021; Hutama et al., 2022; Khade et al., 2023). Our earlier research has found that there is a statistically significant positive correlation between COVID-19 and symptoms like dryness, itchiness, ocular pain, eye swelling, and increased eye discharge. Additionally, there was a significant rise in the percentage of patients with scleritis, iritis, uveitis, and optic neuritis (unpublished article). The etiology of the increased prevalence of ocular disease in people with COVID-19 is not fully understood, and investigating the relationship between COVID-19 and ocular surface microbiome may provide new insights into this phenomenon.

The ocular surface is a complex and important anatomical functional unit of the eye that plays a vital role in vision. It consists of several parts that work together to maintain the health and function of the eye, including the cornea, conjunctiva, lacrimal glands, meibomian gland, tear film, eyelids and the nasolacrimal duct (Cher, 2014; Sridhar, 2018). The ocular surface has many functions, including tear film formation, immune response and environmental protection, and understanding the anatomy and physiology of the ocular surface is essential for treating ocular surface disease and optimizing visual outcomes (Gipson, 2007). The ocular surface microbiome (OSM) is an important component of the ocular surface microenvironment, and plays a significant role in innate and adaptive immunity (Huang et al., 2016). In recent years, there has been a suggestion from the gut-eye axis theory that gut microbiota may impact ocular surface health by controlling IgA secretion, a finding that is critical for maintaining immune homeostasis at the ocular surface in both healthy and diseased conditions (Kugadas et al., 2017). We are aware that the onset and development of various ocular diseases are related to a dysbiosis of ocular microbiota. Such diseases encompass dry eye, conjunctivitis, keratitis, chronic graft-versus-host disease, and SJS, among others (Gomes et al., 2020). Understanding the alterations in the microbial composition of the ocular surface comprehensively and accurately delineating its role in preserving eye well-being is essential for the prevention, detection, and treatment of ocular disorders related to COVID-19.

16S rRNA amplicon sequencing techniques typically target one or more variable regions, utilizing universal primers developed from conserved regions for Polymerase Chain Reaction (PCR) amplification. The amplified DNA is subsequently sequenced and analyzed to identify bacterial species found within the hypervariable regions. Currently, 16S rRNA amplicon sequencing techniques is extensively used to establish a species description, species taxonomy, and phylogenetic relationships. In contrast to the conventional culture-based approach, this method facilitates rapid and precise bacterial identification and amplifies the comprehensiveness of microbiome research. Consequently, it has emerged as the preeminent method for studying microbial diversity (Church et al., 2020).

This study aims to determine the OSM composition of COVID-19 patients using 16S rRNA amplicon sequencing technology and compare results with non-COVID-19 patients and COVID-19 convalescents. From an OSM perspective, the study aims to provide evidence for an increased incidence of ocular disease in COVID-19 patients.

## 2 Materials and methods

This study adhered to the tenets of the Declaration of Helsinki, and all participants received and signed written informed consent. The Ethics Committee of the First Affiliated Hospital of Harbin Medical University approved the study protocol, and registered with China Clinical trial Center (IRB-AF/SC-05/04.0).

### 2.1 Participants

A total of 43 patients who visited the Eye Hospital of First Affiliated Hospital of Harbin Medical University from January to April 2023 were included in this study, and all participants underwent a detailed medical history, ophthalmological examination and SARS-CoV-2 viral gene detection by reverse transcription-polymerase chain reaction (RT-PCR). We then divided all participants into three groups: the control group (C group) consisted of 15 participants who have never had COVID-19, the experimental group (E group) included 15 participants who tested positive for COVID-19, and the COVID-19 recovery period group (R group) comprised 13 participants.

The following criteria were used to include participants: (1) The C group refers to a population that has never had COVID-19. These participants have no clinical manifestations associated with COVID-19 and have negative results on the following microbiological and serological tests: (a) two consecutive negative nucleic acid tests with Ct values ≥35, (b) three consecutive negative antigen test results; (2) The E group refers to a population that shows clinical manifestations related to COVID-19 infection and has positive results on one or more of the following microbiological or serological tests: (a) positive COVID-19 nucleic acid test, or (b) positive COVID-19 antigen test; (3) The R group refers to a population that shows significant improvement in other symptoms and meets any of the following criteria after being infected with the novel coronavirus, indicating the recovery phase: (a) two consecutive negative nucleic acid tests with Ct values ≥35, (b) three consecutive negative antigen test results, (c) a four-fold or higher increase in specific IgG antibodies against COVID-19 during the recovery phase compared to the acute phase, or (d) the fever symptoms have subsided for more than 24 h without using antipyretic drugs after completing 7 days of home isolation; (4) all patients cooperated in completing relevant ophthalmic examinations. Exclusion criteria included: (1) corneal contact lens wear for more than 1 month; (2) history of ocular surgery such as corneal transplantation within 6 months or refractive surgery within 2 years; (3) history of treatment with antibiotics, glucocorticoids and other eye drops within 6 months; (4) ophthalmic diseases that affect the health of the ocular surface (dry eye, conjunctivitis, keratitis, blepharitis, chronic limbal stem cell deficiency, Behcet’s disease, ocular chemical burns, ocular trauma, and so on); (5) systemic diseases affecting the health of the ocular surface (SJS, Sjogren’s syndrome, Parkinson’s disease, rheumatoid arthritis, Grave’s disease, and systemic lupus erythematosus, and so on).

### 2.2 Sample collection

The conjunctival microbial samples were collected from the right eye of each subject using a disposable sterile dry swab. The sterile swab was held horizontally and passed across the length of the superior and inferior tarsal conjunctiva four times, rotating it a quarter turn with every pass. This process was repeated twice for each eye and two air swabs were collected as blank controls (Solomon et al., 2003). The collected swabs were immediately placed in sterile tubes, transported to the laboratory on ice, and stored at −80°C for subsequent testing and DNA extraction. All samples were collected in an ultraviolet-sterilized ophthalmic examination room by the same trained professional ophthalmologist wearing a sterile mask over a sterile to ensure sample consistency.

### 2.3 Total DNA extraction, construction of amplicon library, and sequencing

DNA was extracted using cetyltrimethylammonium bromide (CTAB), and purity and concentration were determined by 2% agarose gel electrophoresis. DNA samples were diluted to 1 ng/μL with sterile water and the V3-V4 region of the bacterial 16S rRNA gene was amplified using primers 341F (5’-CCT AYG GGR BGC ASC AG-3′) and 806R (3′-GGA CTA CNN GGG GTA TCT AAT-5′) (Niem et al., 2020) with a strict control over amplifying cycles. All mixtures were loaded with 15 μL Phusion® High-Fidelity PCR Master Mix (New England Biolabs), 0.2 μM primers and 10 ng genomic DNA template and subjected to PCR. The PCR amplification program was set up as follows: initial denaturation of samples at 98°C for 1 min, followed by 30 cycles of denaturation at 98°C for 10 s, annealing at 50°C for 30 s, and extension at 72°C for 30 s. A final extension step of 5 min at 72°C was added to ensure complete amplification of the target region. An equal volume of 10× loading buffer (contained SYB green) was mixed with the PCR products and electrophoresed on a 2% agarose gel for detection. The mixed PCR products were then purified using Universal DNA Purification Kit (TianGen, China, Catalog #: DP214). Sequencing libraries were prepared using the NEB Next® Ultra™ II FS DNA PCR-free Library Prep Kit (New England Biolabs, United States, Catalog #: E7430L) according to the manufacturer’s recommendations and indexes were added. The library was checked using Qubit and real-time PCR for quantification and Bioanalyzer for size distribution detection. Quantified libraries were pooled and sequenced on Illumina platforms using NovaSeq 6,000 for PE 250, depending on the effective library concentration and the amount of data required.

### 2.4 Sequencing data processing

The data for each sample was separated from the downstream data based on the barcode sequence and PCR amplification primer sequence. The paired-end reads from each sample were merged using FLASH^1^ (Magocˇ and Salzberg, 2011) and the resulting spliced sequences were the raw tags. The spliced raw tags were then rigorously filtered using fastp (Version 0.23.1) software (Bokulich et al., 2013; Chen et al., 2022) to obtain high quality clean tags. The tags were then compared with the species annotation databases^2^ using UCHIME Algorithm (Edgar et al., 2011) to detect chimera sequences, and then the chimera sequences were removed. Then the effective tags were finally obtained.

The previously obtained effective tags were denoised with DADA2 module of the QIIME2 software (Version QIIME2-202006) to obtain the initial amplicon sequence variants (ASVs) (Bokulich et al., 2018). Species annotation was then performed to obtain the phylogenetic relationships of each ASV sequences using QIIME2 software based on the Silva 138.1 database for rapid multiple sequence comparison. Finally, a standard sequence number corresponding to the sample with the fewest sequences was used to normalize the absolute abundance of ASVs.

Alpha diversity was performed based on these derived data such as Chao1, Shannon, and Goods coverage using QIIME2 software. To assess the complexity of the community composition and to compare the differences between samples, beta diversity analyses based on weighted and unweighted unifrac distances were also performed in QIIME2. Non-metric multidimensional scaling (NMDS) analyses, based on the species information contained in samples represented as points on a 2D plane, better reflect the non-linear structure of ecological data (Noval Rivas et al., 2013). To find the biomarkers with a linear discriminant analysis (LDA) threshold of 4, the linear discriminant analysis effect size (LEfSe) software (Version 1.0) was used to perform the LEfSe analysis. Furthermore, it is possible to correlate the annotation results with the corresponding functional databases for functional prediction analyses of the microbial communities across all samples using Phylogenetic Investigation of Communities by Reconstruction of Unobserved States (PICRUSt2) software (Version 2.1.2-b) (Douglas et al., 2020).

### 2.5 Statistical analysis

The statistical analysis was performed with the SPSS v.18.0 statistical software for Windows (SPSS Inc., Chicago, IL). In statistical descriptions, continuous variables are expressed as mean ± standard deviation and categorical variables are expressed as proportions. The Kruskal-Wallis non-parametric test was used to compare whether age differences between the three groups of participants were significant, and the χ2 test was used to compare whether gender differences between the three groups of participants were statistically significant. We used permutational multivariate analysis of variance based on distances (PERMANOVA) (Chen and Zhang, 2021) and analysis of similarity (ANOSIM) (Yang et al., 2019) analyses to test for significant differences in community structure between groups. Statistically significant was defined as a *p*-value below 0.05.

## 3 Results

**Figure 1:**
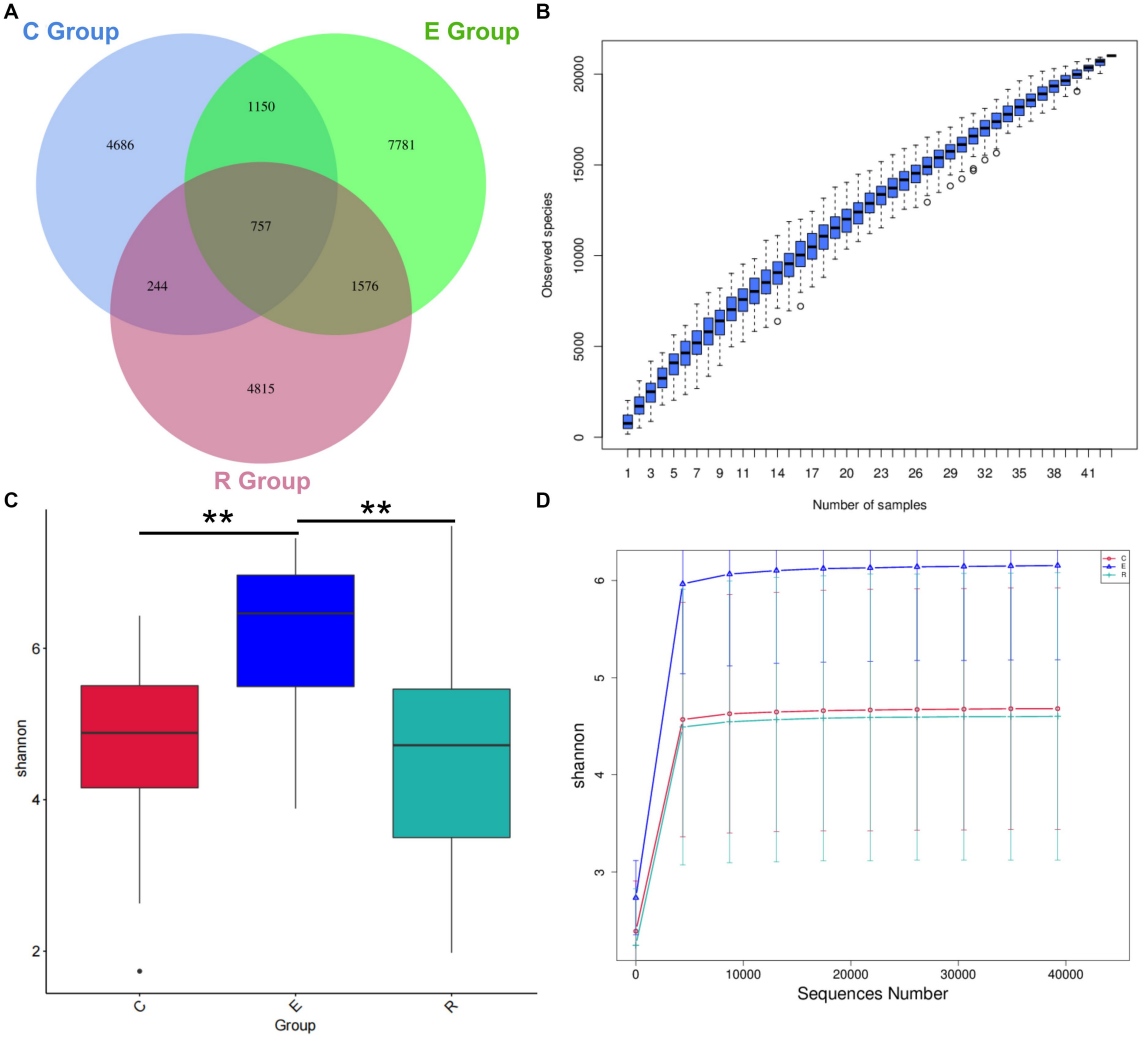
Venn diagram and alpha diversity analysis. **(A)** Venn diagram showing how the AVAs of the Group Members relate to each other. **(B)** Species Accumulation Boxplot shows an increase in species diversity as sample size increases. **(C)** Shannon index box diagram showing significance differences in species diversity between groups, ^*^p < 0.05, ^**^p < 0.01, ^***^p < 0.001. **(D)** Shannon index Rarefaction Curve suggests that the amount of sequencing data is asymptotically reasonable and that more data does not have a significant effect on the alpha diversity index.

A total of 43 patients were invited to participate in this study and Table 1 shows the demographic characteristics of all participants, with 15 in group C (34.9%, range 13–76), 15 in group E (34.9%, range 19–79) and 13 in group R (30.2%, range 14–81). There was no statistically significant difference in age between the groups (H = 0.646, *p* = 0.724), and the male to female ratio was comparable between the groups (*χ*^2^ = 0.23, *p* = 0.893). 16S rRNA sequencing detected a total of 5,128,244 original sequences, with an average of 119,261 original sequences per sample and an average length of 414 bp. A total of 423,747,471 valid sequences were obtained after splicing and filtering out low quality, short sequences and chimeras. After DADA2 noise reduction, a total of 22,222 de-duplication AVAs were obtained. The Venn diagram showed that 4,686, 7,781 and 4,815 characteristic AVAs were detected in the C, E and R groups respectively, while 757 coexisting AVAs were detected (Figure 1A).

**Table 1:** Demographic characteristics: distribution of age, sex.

Features		C group	E group	R group
Sample size (n)		15	15	13
Age (years)	Range	13–76	19–79	14–81
	Mean ± SEM	56.1 ± 4.3	58.7 ± 5.0	55.3 ± 5.7
Sex, n (%)	Male	6 (40.0%)	7 (46.7%)	5 (38.5%)
	Female	9 (60.0%)	8 (53.3%)	8 (61.5%)

SEM, standard error of mean.

### 3.1 Alpha diversity and beta diversity

Alpha diversity can be a reflection of the richness and diversity of microbial communities within a sample through analysis of a single sample. The species accumulation boxplot trend gradually flattened, indicating that the three groups had sufficient sample size and that further increases in sample size would not significantly increase ocular surface microbiota (Figure 1B). In order to correspond to the species diversity and richness of the three sample groups, the Shannon index was chosen. Mean Shannon index for groups C, E and R was 4.68 (IQR = 1.46), 6.15 (IQR = 1.63) and 4.60 (IQR = 2.31), and Shannon index in group E significantly increased compared with groups C and R (*p* < 0.05), indicating that OSM diversity was higher in group E samples (Figure 1C). Similarly, the rarefaction curve tended to flatten, suggesting that the amount of sequencing data was gradual and reasonable, that the species coverage was satisfactory and a larger amount of data would not have a significant effect on the alpha diversity index (Figure 1D). The Chao1 index was used to describe species richness, and the Goods coverage index was used to describe depth of sequencing (Supplementary Figure S1).

**Figure 2:**
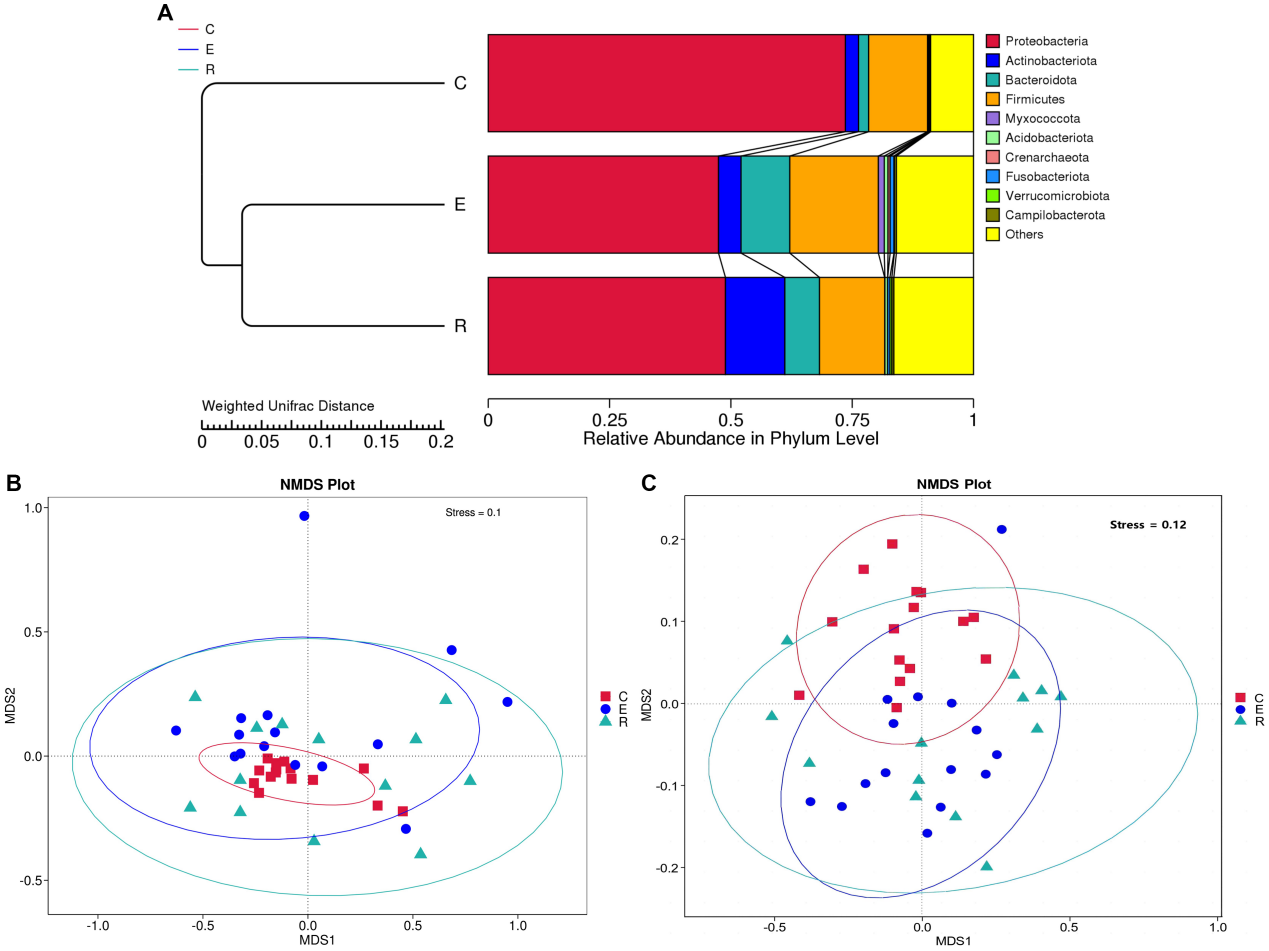
Beta diversity analysis. **(A)** UPGMA cluster analysis. **(B)** NMDS statistics based on the weighted unifrac distance. **(C)** NMDS statistics based on the unweighted unifrac distance, stress values less than 0.2 indicates that the NMDS accurately reflects the degree of variation between samples.

For the comparative analysis of the composition of the ocular surface microbiota between different groups, beta diversity was performed. Unweighted pair-group method with arithmetic mean (UPGMA) cluster analysis, based on the weighted unifrac distance matrix, clustered samples with high similarity in the composition of the ocular surface microbiota (Figure 2A). Group E and R ocular surface microbiota drifted apart and then clustered with group C to form a phylogenetic tree, indicating that group C ocular surface microbiota composition was significantly different from groups E and R. Combined with ANOSIM analysis and ADONIS analysis (Table 2), it was further demonstrated that the OSM remained altered after COVID-19 recovery, similar to that during COVID-19 infection, providing a preliminary explanation for the increased incidence of ocular surface disease caused by COVID-19. Non-metric multi-dimensional scaling (NMDS) statistics based on the weighted unifrac distance (Figure 2B) and unweighted unifrac distance (Figure 2C) showed that the bacterial community was classified into three groups, with stress values of 0.1 and 0.12 respectively, indicating that NMDS can accurately reflect the degree of difference between samples.

**Table 2:** ANOSIM and PERMANOVA analysis of the groups.

Statistical tests		C-E	C-R	E-R
ANOSIM	*R*-value	0.36912	0.30713	0.10736
	*P*-value	0.001^***^	0.001^***^	0.028^*^
PERMANOVA	*R* ^2^	0.16905	0.14390	0.05588
	*P*-value	0.001^***^	0.001^***^	0.073

R-value: between (−1, 1), R-value greater than 0 indicates that the difference between groups is greater than the difference within groups, R-value less than 0 indicates that the difference within groups is greater than the difference between groups; R (Chen et al., 2020): indicates the degree to which the sample variance is explained by different groups, i.e., the ratio of subgroup variance to total variance; the higher the R2, the greater the degree to which the subgroups explain the variance; *p*-value: less than 0.05 means the test is highly reliable, ^*^ < 0.05, ^**^ < 0.01, ^***^ < 0.001.

### 3.2 Microbial community composition and structure

**Figure 3:**
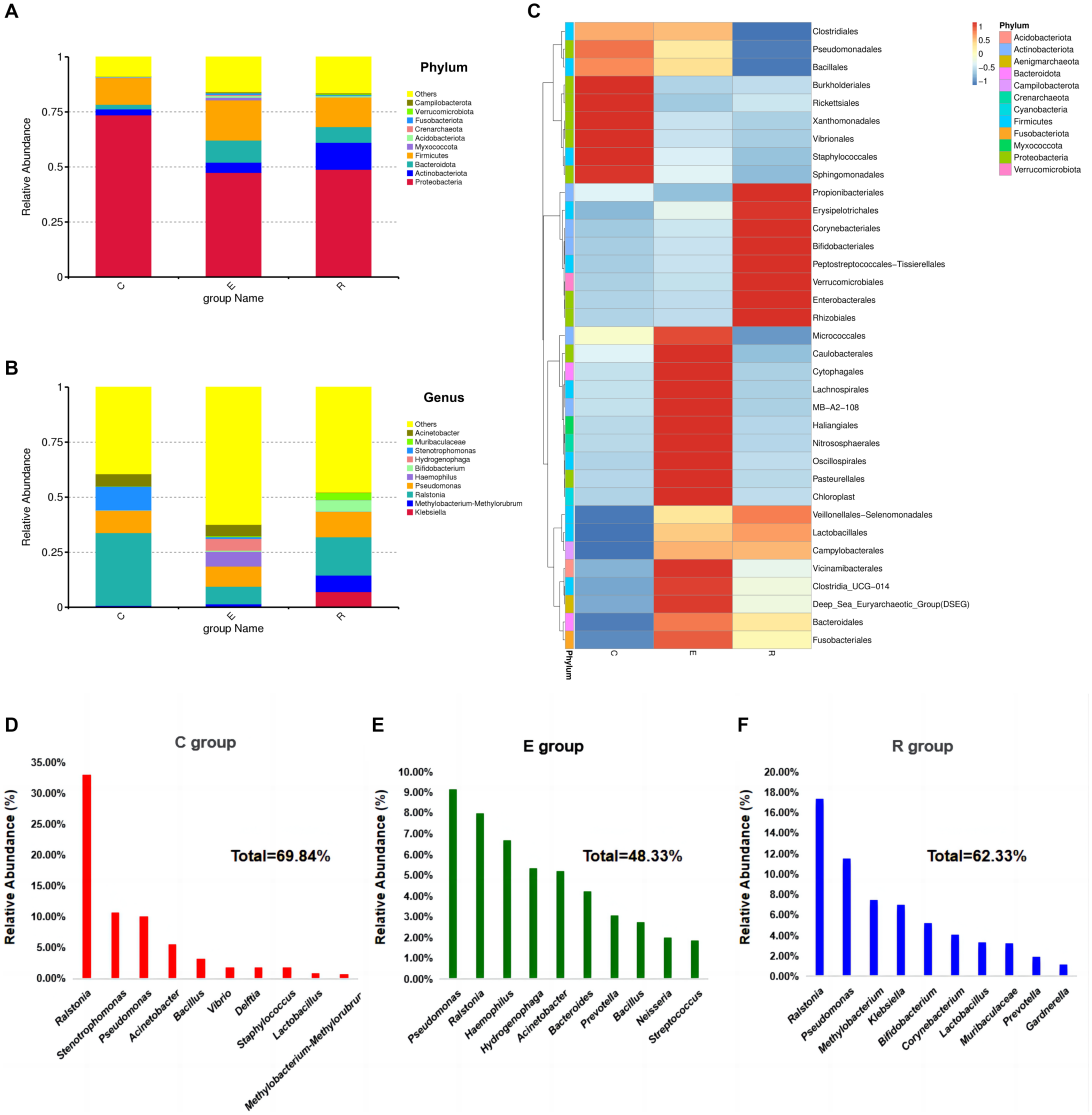
Relative abundances of dominant phyla in samples. **(A)** Accumulative histogram of species abundance at the phylum level, other bacterial abundances in groups C, E and R were 8.84, 15.89 and 16.42%. **(B)** Accumulative histogram of species abundance at the genus level, other bacterial abundances in groups C, E and R were 39.41, 62.42, 47.78%. **(C)** Heatmap of microbial composition cluster analysis at genus level in each group. **(D-F)** Composition and proportions of the top 10 species ranked by relative abundance at genus level in groups C, E and R.

A total of 52 species annotated at the phylum level and 1,521 species annotated at the genus level resulted from the species annotation of all ASA sequences. At the phylum level, the OSM of all three groups was dominated by *Proteobacteria*, *Actinobacteriota*, *Bacteroidota* and *Firmicutes*, accounting for more than 80% of the total abundance (Figure 3A). When analyzing the *Proteobacteria*, the group E (47.46%) and the group R (48.88%) were significantly less abundant compared to the group C (73.59%). The analysis for *Actinobacteriota* showed an increase in the abundance of this phylum in group E (4.63%) and group R (12.24%) compared to group C (2.72%). In the case of *Bacteroidota*, the analysis revealed a significant increase in abundance in group E (10.05%) and group R (7.14%) compared to group C (2.08%). For *Firmicutes*, the analysis showed that there was less difference between the C group (12.14%) and the R group (14.43%), while the E group (18.25%) had a higher abundance of this phylum.

At the genus level, the composition of the ocular surface microbiota differed significantly between the three groups (Figure 3B), and the clustering heatmap of species abundance showed the top 35 genus in terms of species richness, providing the foundation to confirm the disruption of the ocular surface microbiota after COVID-19 infection (Figure 3C). *Ralstonia* (33.06%), *Stenotrophomonas* (10.73%), *Pseudomonas* (10.05%), *Acinetobacter* (5.57%) and *Bacillus* (3.30%) were the five most common genus in group C (Figure 3D). In group E, the five most abundant genus were *Pseudomonas* (9.17%), *Ralstonia* (7.98%), *Haemophilus* (6.70%), *Hydrogenophaga* (5.35%), *Acinetobacter* (5.20%) (Figure 3E). The five most abundant genus in the R group were *Ralstonia* (17.39%), *Pseudomonas* (11.53%), *Methylobacterium-Methylorubrum* (7.49%), *Klebsiella* (7.05%), *Bifidobacterium* (5.17%) (Figure 3F).

**Figure 4:**
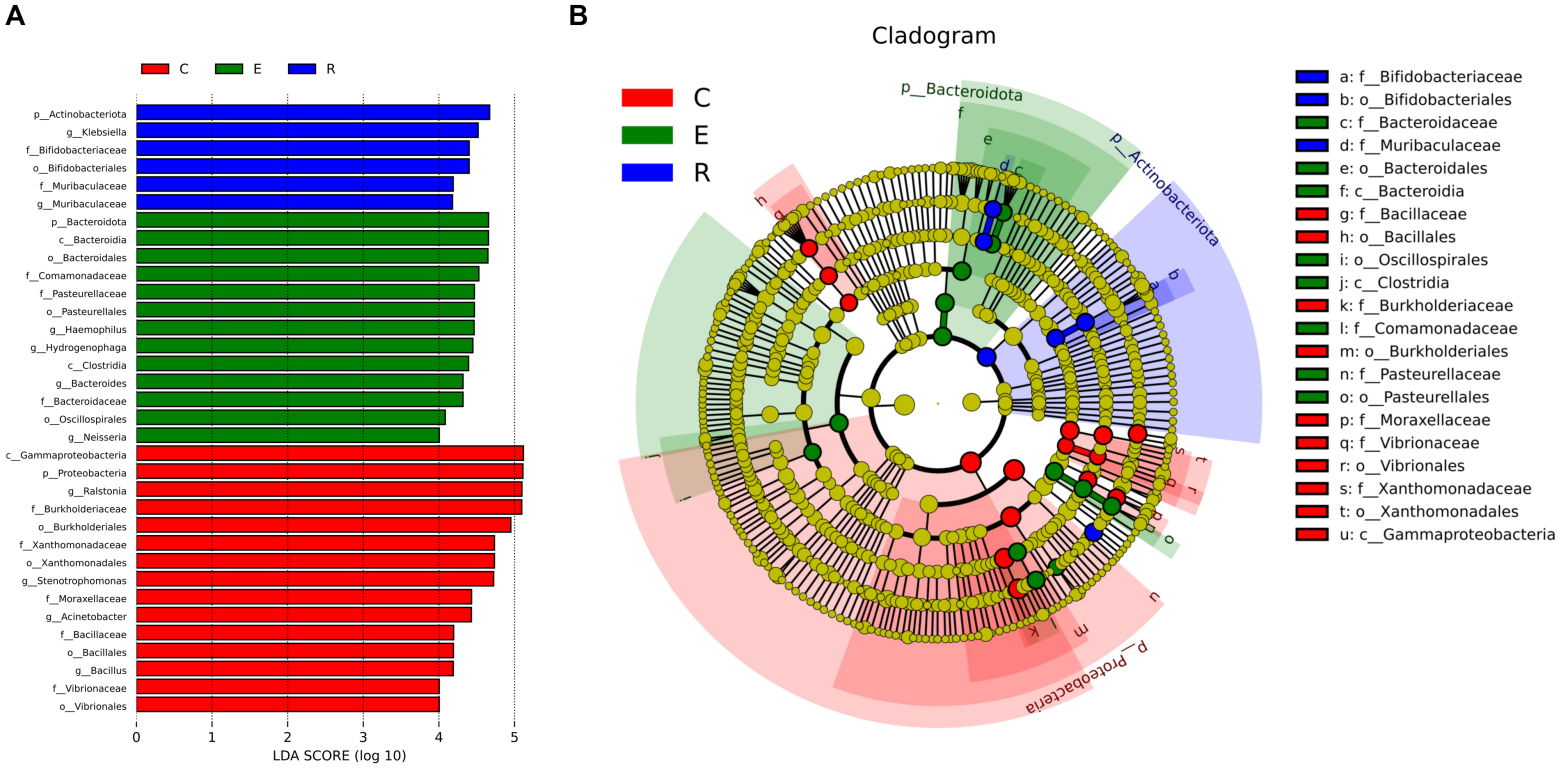
Biomarkers with statistically significant differences between the three groups. **(A)** The histogram of LDA value distribution. **(B)** The evolutionary branching diagram. LEfSe plot of taxonomic biomarkers identified in the ocular surface microbiome of patients and controls. LDA scores are effect size estimates for a particular taxonomic marker in a specific group, with values interpreted as the relative magnitude of abundance compared to the other group.

Following analysis of the LEfSe between the three groups, the histograms of the distribution of LDA scores (Figure 4A) and the evolution tree (Figure 4B) revealed that a total of 34 biomarkers showed significant differences between the three groups (LDA scores >4). At the genus level, the statistically significant biomarkers in group C were *Ralstonia*, *Stenotrophomonas*, *Acinetobacter*, *Bacillales*. Group E included *Haemophilus*, *Hydrogenophaga*, *Bacteroides* and *Neisseria*, and group R included *Klebsiella* and *Muribacula*.

### 3.3 Predicted functional profiling of microbial communities

The biological functions of microbial communities were predicted using PICRUSt2, and functional annotations were performed on the basis of the 16S rRNA amplicon sequencing data using the Kyoto Encyclopedia of Genes and Genomes (KEGG) database. The top 10 most abundant functional information is shown in the relative function abundance accumulative histogram, which provides visual representation of the proportion of each function for each group, where signaling and cellular processes, lipid metabolism, amino acid metabolism and genetic information processing were the ubiquitous biological functions in all groups (Supplementary Figure S2A). We then selected the 35 most abundant functions and their abundance information in each group to heat map and cluster at different functional levels, which revealed unique functional annotation information among the groups. Among these, group E was more prominent in terms of membrane transport, cellular community - prokaryotes functions, while group R was more unique in cell growth and death, and fatty acid biosynthesis and degradation (Supplementary Figure S2B).

## 4 Discussion

Many studies have now shown that the OSM plays a key role in maintaining the homeostasis of the ocular surface microenvironment, despite its relatively stable species composition and low diversity and abundance in the physiological state, especially the proposed gut-eye axis, which provides a solid theoretical basis for OSM-related research (Bu et al., 2021). Healthy human ocular surfaces harbor a symbiotic bacterial community, with the most abundant phylum being *Proteobacteria*, *Actinobacteria*, and *Firmicuts*, with the most abundant genus being *Corynebacterium*, *Pseudomonas*, and *Staphylococcus* (Zilliox et al., 2020). In pathological states, the OSM is susceptible to perturbations from external factors such as diet, antibiotics and infection, leading to ecological imbalances, and disruption of this homeostasis can promote the growth and invasion of pathogenic species, leading to changes in the immune status of the ocular surface (Kugadas et al., 2016; Berg et al., 2020). We therefore collected and analysed conjunctival sac swab specimens from 43 participants to investigate the effect of SARS-CoV-2 infection on OSM and concluded that COVID-19 patients’ OSM composition differed significantly from that of the normal population and this change persisted for up to 2 months in COVID-19 convalescent period.

In recent years, numerous studies have been conducted to explore how the COVID-19 affects the composition and diversity of the microbiota. The upper respiratory tract is the initial site of infection for SARS-CoV-2, and the microbiota may regulate viral replication by influencing the local immune environment (Weiss et al., 2020; Zou et al., 2020). There is growing evidence that COVID-19 affects the upper respiratory tract microbiome, particularly in the nasopharynx. While study results have varied, a consistent finding is the significant change in the relative abundance of the bacterial genus *Corynebacterium* in the nasopharynx of individuals infected with SARS-CoV-2 (Candel et al., 2023). This observation is robust and has become a key area of research in several studies. Evidence suggests ocular surface microbes can affect the nasopharynx via the nasolacrimal system (Spencer et al., 2021), and changes in ocular surface and nasopharyngeal microbiome in allergic rhinoconjuctivitis are consistent, but the mechanisms by which they might influence each other require further investigation (Yau et al., 2019).

Additionally, the microbiota in the upper respiratory tract can spread to the lower respiratory tract, including the lungs, and has been associated with adverse outcomes such as acute lung injury in COVID-19 patients (Merenstein et al., 2022). The severity of COVID-19 disease varies, and patients exhibit significant differences in the composition of the upper respiratory tract microbiota. *Streptococcus* is significantly enriched in individuals with a favorable prognosis and shows good temporal stability. However, in severe patients, the microbiota composition differs significantly from that of healthy individuals and is more prone to causing secondary infections (Ren et al., 2021).

Although COVID-19 is considered a respiratory system disease, its gastrointestinal symptoms should not be overlooked. Yun Kit Yeoh et al. conducted metagenomic sequencing of fecal samples from 100 COVID-19 patients and found that there was a significant decrease in microbial populations such as *Faecalibacterium prausnitzii*, *Eubacterium rectale*, and *Bifidobacteria* in the COVID-19 patients. These microbial relative abundance remained at lower levels even 30 days after disease resolution, suggesting that the gut microbiota may play a role in influencing the severity of COVID-19 by affecting the host immune response (Yeoh et al., 2021). There is currently limited research on the impact of COVID-19 on the ocular microbiome. Our study aims to address this gap and provide theoretical support for a deeper understanding of the pathogenesis of COVID-19-related ocular diseases based on the ocular microbiome.

In our study, we observed higher alpha and beta diversities in groups E and R compared to group C, indicating a richer microbial composition in the samples, which is usually associated with disease. The microbial composition of the E and R groups demonstrated greater similarity, indicating the persistent effects of COVID-19 on ocular health. For a better understanding of OSM, the microbial composition needs to be analysed at phylum and genus level. At the phylum level, the composition and abundance of the strains detected in Group C was consistent with existing reports of OSMs in the healthy state, with a predominance of *Proteobacteria*, *Actinobacteriota*, and *Firmicutes*, demonstrating the accuracy and reliability of the assay (Huang et al., 2016; Gomes et al., 2020; Zilliox et al., 2020). In groups E and R, *Proteobacteria* decreased significantly, while the remaining three phylum increased significantly in species richness, with *Actinobacteriota* and *Bacteroidota* showing the most significant increase. This usually indicates a disturbance in the homeostatic balance of the OSM and an increase in susceptibility to disease.

At the genus level, there were significant differences in OSM composition and proportions between the three groups, and OSM species diversity was richer in samples from groups E and R compared to group C, *Pseudomonas* emerges as new dominant group associated with increased susceptibility to keratitis (Cavuoto et al., 2021). In addition, a significant decrease in the abundance of other species, particularly *Staphylococcus*, has been shown to correlate significantly with conditions such as meibomian gland dysfunction(MGD) (Zhao et al., 2020; Ozkan et al., 2023). *Haemophilus*, *Streptococcus* were elevated in group E, associated with diseases such as conjunctivitis, trachoma and SJS (Zhou et al., 2014; Kittipibul et al., 2020; Song et al., 2022). *Corynebacterium*, *Klebsiella*, *Lactobacillus*, and *Prevotella* were elevated in group R, associated with diseases such as dry eye, blepharitis, trachoma, and SJS (Kittipibul et al., 2020; Andersson et al., 2021; Fu et al., 2022). This finding provides a solid theoretical basis for explaining the phenomenon from an OSM perspective, which is consistent with our previous finding of an increased incidence of associated ocular disease associated with SARS-CoV-2 infection. Simultaneously, we identified representative biomarkers within each group. In Group E, notable biomarkers such as *Haemophilus*, *Bacteroides*, and *Neisseria* were significantly associated with heightened susceptibility to conjunctivitis in patients during the acute phase of COVID-19 (Song et al., 2022). Conversely, Group R exhibited associations, particularly with *Klebsiella*, indicating a significant correlation with increased susceptibility to dry eye in patients during the recovery phase of COVID-19 (Jung et al., 2022).

At present, SARS-CoV-2 infections persist and remain a significant threat to human health. As we hypothesized, conjunctivitis, an early clinical manifestation of SARS-CoV-2 infection, has been found to be specifically linked to changes in the OSM only during the acute phase of COVID-19. Once the acute inflammation subsides, the OSM gradually returns to its stable state, leading to a reduction in ocular pain, photophobia, and lacrimation. However, the reasons why changes in the microbial composition associated with chronic ocular surface diseases such as dry eye, blepharitis, MGD and SJS can persist long after COVID-19 recovery are worthy of further investigation. Firstly, COVID-19 is a systemic “cytokine storm.” By analysing the clinical data of 3,933 COVID-19 patients, Wang J et al. identified elevated levels of several cytokines, including IL-6, IL-1β, IL-10, TNF-α (Wang et al., 2020). These cytokines can also be recruited on the ocular surface, altering the ocular immune status and resulting in microbial imbalance. This further exacerbates inflammatory reactions, ultimately leading to a self-perpetuating cycle that leaves chronic ocular inflammation continuously impacting patients’ health. Secondly, mask use was greatly increased during COVID-19, and the upward flow of air toward the eyes when wearing an unfitted mask can cause changes in temperature and humidity at the ocular surface (Burgos-Blasco et al., 2023). Many studies have shown that long-term mask wear can lead to a destabilization of the tear film, an increase in inflammatory factors such as IL-1β, IL-33 and IFN-β, and an increase in corneal dendritic cell density, these changes affect the environment in which ocular commensal bacteria live and ultimately lead to diseases such as dry eye, MGD, etc (D’Souza et al., 2022). Thirdly, while the COVID-19 vaccine itself does not induce illness, its adjuvant component may provoke an immune reaction against microbial pathogens. There have been numerous case reports indicating that COVID-19 vaccination can cause or worsen ocular inflammatory conditions such as conjunctivitis, scleritis, uveitis, and optic neuritis, which may be attributed to altered metabolism of spike proteins and the activation of Toll-like receptors (Ng et al., 2022). Furthermore, it is possible that ocular surface health may be affected by the increased frequency of video display terminals and the altered mental health status of patients during SARS-CoV-2 infection, although the specific mechanisms require further investigation (Santomauro et al., 2021; Fjærvoll et al., 2022). In order to promote eye health, we advise patients to follow the recommended measures. Firstly, it is advisable to refrain from rubbing the eyes as doing so can adversely affect the OSM. Secondly, it is recommended to utilize suitable eye masks to maintain a stable eye environment. Lastly, if patients encounter any clinical symptoms pertaining to eyes, it is crucial to promptly seek medical attention.

However, this study also has some limitations. Firstly, there are limitations to the 16S rRNA sequencing technology, the lack of 16S sequence data currently available is a serious limitation to the comprehensive identification of clinical pathogens (Huang et al., 2016). Secondly, we acknowledge that the composition of microbial communities at the genus level vary from previous research findings. This discrepancy could be attributed to the limited sample sizes, which may not accurately represent the general population. Moreover, variations in microbial composition can also arise from different living environments and geographic locations (Deng et al., 2020). Finally, OSM is not limited to bacteria, but should also include fungi, viruses and other microorganisms, this study is not yet representative of the full spectrum of OSM in COVID-19 (Wang et al., 2020). To overcome these limitations, our future research will aim to expand the sample size and utilize metagenomic profiling techniques to monitor changes in the microbial composition of the ocular surface, including bacteria, viruses, and fungi. By doing so, we hope to further investigate the underlying reasons for the increased incidence of ocular diseases associated with SARS-CoV-2 infections.

In conclusion, the present study is the first to characterize the ocular surface microbiome of COVID-19 patients using 16S rRNA amplicon sequencing technology and demonstrated the uniqueness of the microbial composition, providing a theoretical basis to explore the mechanism of increased ocular disease morbidity after SARS-CoV-2 infection from the perspective of OSM. In the future, it will be crucial to conduct in-depth investigations into the relationships among ocular diseases, OSM, and immune responses in COVID-19 patients during long-term follow-up periods of 1 year or more. We anticipate that conducting a more comprehensive examination of these connections could indeed provide innovative, feasible and evidence-based strategies for preventing and managing ocular surface diseases associated with COVID-19.

## Data availability statement

The data presented in the study are deposited in the NCBI Sequence Read Archive repository, accession number PRJNA1131594.

## Ethics statement

The studies involving humans were approved by The Ethics Committee of the First Affiliated Hospital of Harbin Medical University. The studies were conducted in accordance with the local legislation and institutional requirements. Written informed consent for participation in this study was provided by the participants’ legal guardians/next of kin.

## Author contributions

JL: Data curation, Methodology, Writing – original draft, Writing – review & editing. JW: Methodology, Writing – review & editing. JF: Conceptualization, Investigation, Writing – review & editing. RZ: Software, Supervision, Validation, Writing – review & editing. YG: Investigation, Methodology, Writing – review & editing. YD: Project administration, Writing – review & editing. HZ: Funding acquisition, Resources, Writing – review & editing. XJ: Conceptualization, Writing – review & editing.

## Footnotes

^1^version 1.2.11, http://ccb.jhu.edu/software/FLASH/

^2^Silva database https://www.arb-silva.de/ for 16S/18S, unite database https://unite.ut.ee/ for ITS

## References

[ref1] Aggarwal K. Agarwal A. Jaiswal N. Dahiya N. Ahuja A. Mahajan S.. (2020). Ocular surface manifestations of coronavirus disease 2019 (COVID-19): a systematic review and meta-analysis. PLoS One 15:e0241661. doi: 10.1371/journal.pone.0241661 33151999 PMC7643964

[ref2] Andersson J. Vogt J. K. Dalgaard M. D. Pedersen O. Holmgaard K. Heegaard S. (2021). Ocular surface microbiota in patients with aqueous tear-deficient dry eye. Ocul. Surf. 19, 210–217. doi: 10.1016/j.jtos.2020.09.003, PMID: 32931939

[ref3] Azzolini C. Donati S. Premi E. Baj A. Siracusa C. Genoni A.. (2021). SARS-CoV-2 on ocular surfaces in a cohort of patients with COVID-19 from the Lombardy region, Italy. JAMA Ophthalmol. 139, 956–963. doi: 10.1001/jamaophthalmol.2020.5464, PMID: 33662099 PMC7934077

[ref4] Berg G. Rybakova D. Fischer D. Cernava T. Vergès M. C. C. Charles T.. (2020). Microbiome definition re-visited: old concepts and new challenges. Microbiome 8:103. doi: 10.1186/s40168-020-00875-0, PMID: 32605663 PMC7329523

[ref5] Bokulich N. A. Kaehler B. D. Rideout J. R. Dillon M. Bolyen E. Knight R.. (2018). Optimizing taxonomic classification of marker-gene amplicon sequences with QIIME 2's q2-feature-classifier plugin. Microbiome 6:90. doi: 10.1186/s40168-018-0470-z, PMID: 29773078 PMC5956843

[ref6] Bokulich N. A. Subramanian S. Faith J. J. Gevers D. Gordon J. I. Knight R.. (2013). Quality-filtering vastly improves diversity estimates from Illumina amplicon sequencing. Nat. Methods 10, 57–59. doi: 10.1038/nmeth.2276, PMID: 23202435 PMC3531572

[ref7] Bu Y. Chan Y. K. Wong H. L. Poon S. H. L. Lo A. C. Y. Shih K. C.. (2021). A review of the impact of alterations in gut microbiome on the Immunopathogenesis of ocular diseases. J. Clin. Med. 10:4694. doi: 10.3390/jcm10204694, PMID: 34682816 PMC8541376

[ref8] Burgos-Blasco B. Arriola-Villalobos P. Fernandez-Vigo J. I. Oribio-Quinto C. Ariño-Gutierrez M. Diaz-Valle D.. (2023). Face mask use and effects on the ocular surface health: a comprehensive review. Ocul. Surf. 27, 56–66. doi: 10.1016/j.jtos.2022.12.006, PMID: 36577463 PMC9789923

[ref9] Candel S. Tyrkalska S. D. Álvarez-Santacruz C. Mulero V. (2023). The nasopharyngeal microbiome in COVID-19. Emerg. Microbes Infect. 12:e2165970. doi: 10.1080/22221751.2023.2165970, PMID: 36606725 PMC9869994

[ref10] Cavuoto K. M. Galor A. Banerjee S. (2021). Ocular surface microbiome alterations are found in both eyes of individuals with unilateral infectious keratitis. Transl. Vis. Sci. Technol. 10:19. doi: 10.1167/tvst.10.2.19, PMID: 34003904 PMC7884290

[ref11] Chen L. Deng C. Chen X. Zhang X. Chen B. Yu H.. (2020). Ocular manifestations and clinical characteristics of 535 cases of COVID-19 in Wuhan, China: a cross-sectional study. Acta Ophthalmol. (Copenh) 98, e951–e959. doi: 10.1111/aos.14472, PMID: 32421258 PMC7276826

[ref12] Chen C. Liao J. Xia Y. Liu X. Jones R. Haran J.. (2022). Gut microbiota regulate Alzheimer’s disease pathologies and cognitive disorders via PUFA-associated neuroinflammation. Gut 71, 2233–2252. doi: 10.1136/gutjnl-2021-326269, PMID: 35017199 PMC10720732

[ref13] Chen J. Zhang X. (2021). D-MANOVA: fast distance-based multivariate analysis of variance for large-scale microbiome association studies. Schwartz R, editor. Bioinformatics 38, 286–288. doi: 10.1093/bioinformatics/btab498, PMID: 34255026 PMC8696110

[ref14] Cher I. (2014). Ocular surface concepts: development and citation. Ocul. Surf. 12, 10–13. doi: 10.1016/j.jtos.2013.10.004, PMID: 24439042

[ref15] Church D. L. Cerutti L. Gürtler A. Griener T. Zelazny A. Emler S. (2020). Performance and application of 16S rRNA gene cycle sequencing for routine identification of Bacteria in the clinical microbiology laboratory. Clin. Microbiol. Rev. 33, e00053–e00019. doi: 10.1128/CMR.00053-19 32907806 PMC7484979

[ref16] D’Souza S. Vaidya T. Nair A. P. Shetty R. Kumar N. R. Bisht A.. (2022). Altered ocular surface health status and tear film immune profile due to prolonged daily mask Wear in health care workers. Biomedicines 10:1160. doi: 10.3390/biomedicines10051160, PMID: 35625896 PMC9139140

[ref17] Deng Y. Wen X. Hu X. Zou Y. Zhao C. Chen X.. (2020). Geographic difference shaped human ocular surface metagenome of young Han Chinese from Beijing, Wenzhou, and Guangzhou cities. Investig. Opthalmol. Vis. Sci. 61:47. doi: 10.1167/iovs.61.2.47, PMID: 32106294 PMC7329964

[ref18] Douglas G. M. Maffei V. J. Zaneveld J. R. Yurgel S. N. Brown J. R. Taylor C. M.. (2020). PICRUSt2 for prediction of metagenome functions. Nat. Biotechnol. 38, 685–688. doi: 10.1038/s41587-020-0548-6, PMID: 32483366 PMC7365738

[ref19] Edgar R. C. Haas B. J. Clemente J. C. Quince C. Knight R. (2011). UCHIME improves sensitivity and speed of chimera detection. Bioinformatics 27, 2194–2200. doi: 10.1093/bioinformatics/btr381, PMID: 21700674 PMC3150044

[ref20] Fjærvoll H. Fjærvoll K. Magno M. Moschowits E. Vehof J. Dartt D. A.. (2022). The association between visual display terminal use and dry eye: a review. Acta Ophthalmol. 100, 357–375. doi: 10.1111/aos.15049, PMID: 34697901

[ref21] Fu Y. Wu J. Wang D. Li T. Shi X. Li L.. (2022). Metagenomic profiling of ocular surface microbiome changes in Demodex blepharitis patients. Front. Cell. Infect. Microbiol. 12:922753. doi: 10.3389/fcimb.2022.922753, PMID: 35937693 PMC9354880

[ref22] Gipson I. K. (2007). The ocular surface: the challenge to enable and protect vision: the Friedenwald lecture. Invest. Ophthalmol. Vis. Sci. 48, 4391–4398. doi: 10.1167/iovs.07-0770 PMC2886589 17898256

[ref23] Gomes J. Á. P. Frizon L. Demeda V. F. (2020). Ocular surface microbiome in health and disease. Asia Pac. J. Ophthalmol. 9, 505–511. doi: 10.1097/APO.0000000000000330, PMID: 33323705

[ref24] Huang Y. Yang B. Li W. (2016). Defining the normal core microbiome of conjunctival microbial communities. Clin. Microbiol. Infect. 22, 643.e7–643.e12. doi: 10.1016/j.cmi.2016.04.008, PMID: 27102141

[ref25] Hutama S. A. Alkaff F. F. Intan R. E. Maharani C. D. Indriaswati L. Zuhria I. (2022). Recurrent keratoconjunctivitis as the sole manifestation of COVID-19 infection: a case report. Eur. J. Ophthalmol. 32, NP17–NP21. doi: 10.1177/11206721211006583 33781126 PMC9294609

[ref26] Jung I. Yoon J. S. Ko B. Y. (2022). Microbiologic analysis of removed silicone Punctal plugs in dry eye patients. J. Clin. Med. 11:2326. doi: 10.3390/jcm11092326, PMID: 35566452 PMC9103617

[ref27] Khade P. Shah A. Kharkar V. (2023). Stevens-Johnson syndrome in adult patient secondary to COVID-19 infection: case report. JMIR Dermatol. 6:e45062. doi: 10.2196/45062, PMID: 37632918 PMC10335157

[ref28] Kittipibul T. Puangsricharern V. Chatsuwan T. (2020). Comparison of the ocular microbiome between chronic Stevens-Johnson syndrome patients and healthy subjects. Sci. Rep. 10:4353. doi: 10.1038/s41598-020-60794-w, PMID: 32152391 PMC7062716

[ref29] Kugadas A. Christiansen S. H. Sankaranarayanan S. Surana N. K. Gauguet S. Kunz R.. (2016). Impact of microbiota on resistance to ocular *Pseudomonas aeruginosa*-induced keratitis. PLoS Pathog. 12:e1005855. doi: 10.1371/journal.ppat.1005855 27658245 PMC5033354

[ref30] Kugadas A. Wright Q. Geddes-McAlister J. Gadjeva M. (2017). Role of microbiota in strengthening ocular mucosal barrier function through secretory IgA. Investig. Opthalmol. Vis. Sci. 58, 4593–4600. doi: 10.1167/iovs.17-22119, PMID: 28892827 PMC5595225

[ref31] Lima L. C. D. F. Moraes Junior H. V. Moraes H. M. V. (2021). COVID-19 ocular manifestations in the early phase of disease. Ocul. Immunol. Inflamm. 29, 666–668. doi: 10.1080/09273948.2021.1887278, PMID: 34242138

[ref32] Magocˇ T. Salzberg S. L. (2011). FLASH: fast length adjustment of short reads to improve genome assemblies. Bioinformatics 27, 2957–2963. doi: 10.1093/bioinformatics/btr507, PMID: 21903629 PMC3198573

[ref33] Merenstein C. Bushman F. D. Collman R. G. (2022). Alterations in the respiratory tract microbiome in COVID-19: current observations and potential significance. Microbiome 10:165. doi: 10.1186/s40168-022-01342-8, PMID: 36195943 PMC9532226

[ref34] Ng X. L. Betzler B. K. Ng S. Chee S. P. Rajamani L. Singhal A.. (2022). The eye of the storm: COVID-19 vaccination and the eye. Ophthalmol. Ther. 11, 81–100. doi: 10.1007/s40123-021-00415-5, PMID: 34914035 PMC8675299

[ref35] Niem J. M. Billones-Baaijens R. Stodart B. Savocchia S. (2020). Diversity profiling of grapevine microbial Endosphere and antagonistic potential of endophytic Pseudomonas against grapevine trunk diseases. Front. Microbiol. 11:477. doi: 10.3389/fmicb.2020.00477, PMID: 32273871 PMC7113392

[ref36] Noval Rivas M. Burton O. T. Wise P. Zhang Y. Hobson S. A. Garcia Lloret M.. (2013). A microbiota signature associated with experimental food allergy promotes allergic sensitization and anaphylaxis. J. Allergy Clin. Immunol. 131, 201–212. doi: 10.1016/j.jaci.2012.10.026, PMID: 23201093 PMC3860814

[ref37] Ozkan J. Majzoub M. E. Coroneo M. Thomas T. Willcox M. (2023). Ocular microbiome changes in dry eye disease and meibomian gland dysfunction. Exp. Eye Res. 235:109615. doi: 10.1016/j.exer.2023.109615, PMID: 37586456

[ref38] Ren L. Wang Y. Zhong J. Li X. Xiao Y. Li J.. (2021). Dynamics of the upper respiratory tract microbiota and its association with mortality in COVID-19. Am. J. Respir. Crit. Care Med. 204, 1379–1390. doi: 10.1164/rccm.202103-0814OC, PMID: 34534435 PMC8865718

[ref39] Saldanha I. J. Petris R. Makara M. Channa P. Akpek E. K. (2021). Impact of the COVID-19 pandemic on eye strain and dry eye symptoms. Ocul. Surf. 22, 38–46. doi: 10.1016/j.jtos.2021.06.004, PMID: 34133976 PMC8462938

[ref40] Santomauro D. F. Mantilla Herrera A. M. Shadid J. Zheng P. Ashbaugh C. Pigott D. M.. (2021). Global prevalence and burden of depressive and anxiety disorders in 204 countries and territories in 2020 due to the COVID-19 pandemic. Lancet 398, 1700–1712. doi: 10.1016/S0140-6736(21)02143-7, PMID: 34634250 PMC8500697

[ref41] Solomon A. W. Holland M. J. Burton M. J. West S. K. Alexander N. D. Aguirre A.. (2003). Strategies for control of trachoma: observational study with quantitative PCR. Lancet 362, 198–204. doi: 10.1016/S0140-6736(03)13909-8, PMID: 12885481

[ref42] Song H. Xiao K. Min H. Chen Z. Long Q. (2022). Characterization of conjunctival sac microbiome from patients with allergic conjunctivitis. J. Clin. Med. 11:1130. doi: 10.3390/jcm11041130, PMID: 35207407 PMC8875969

[ref43] Spencer S. K. R. Francis I. C. Coroneo M. T. (2021). Spontaneous face- and eye-touching: infection risk versus potential microbiome gain. Ocul. Surf. 21, 64–65. doi: 10.1016/j.jtos.2021.04.008, PMID: 33940169 PMC8086376

[ref44] Sridhar M. S. (2018). Anatomy of cornea and ocular surface. Indian J. Ophthalmol. 66, 190–194. doi: 10.4103/ijo.IJO_646_17, PMID: 29380756 PMC5819093

[ref45] Wang Y. Chen H. Xia T. Huang Y. (2020). Characterization of fungal microbiota on normal ocular surface of humans. Clin. Microbiol. Infect. 26, 123.e9–123.e13. doi: 10.1016/j.cmi.2019.05.011, PMID: 31128284

[ref46] Wang J. Jiang M. Chen X. Montaner L. J. (2020). Cytokine storm and leukocyte changes in mild versus severe SARS-CoV-2 infection: review of 3939 COVID-19 patients in China and emerging pathogenesis and therapy concepts. J. Leukoc. Biol. 108, 17–41. doi: 10.1002/JLB.3COVR0520-272R 32534467 PMC7323250

[ref47] Weiss A. Jellingsø M. Sommer M. O. A. (2020). Spatial and temporal dynamics of SARS-CoV-2 in COVID-19 patients: a systematic review and meta-analysis. EBioMedicine 58:102916. doi: 10.1016/j.ebiom.2020.102916, PMID: 32711256 PMC7374142

[ref48] World Health Organization. (2023) *WHO Coronavirus (COVID-19) Dashboard*. Geneva: World Health Organization. https://covid19.who.int/data (Accessed November 4, 2023).

[ref49] Yang R. H. Bao D. P. Guo T. Li Y. Ji G. Y. Ji K. P.. (2019). Bacterial profiling and dynamic succession analysis of Phlebopus portentosus casing soil using MiSeq sequencing. Front. Microbiol. 10:1927. doi: 10.3389/fmicb.2019.01927, PMID: 31507552 PMC6716355

[ref50] Yau J. W. Hou J. Tsui S. K. W. Leung T. F. Cheng N. S. Yam J. C.. (2019). Characterization of ocular and nasopharyngeal microbiome in allergic rhinoconjunctivitis. Pediatr. Allergy Immunol. 30, 624–631. doi: 10.1111/pai.13088, PMID: 31132163

[ref51] Yeoh Y. K. Zuo T. Lui G. C. Y. Zhang F. Liu Q. Li A. Y.. (2021). Gut microbiota composition reflects disease severity and dysfunctional immune responses in patients with COVID-19. Gut 70, 698–706. doi: 10.1136/gutjnl-2020-323020, PMID: 33431578 PMC7804842

[ref52] Zhao F. Zhang D. Ge C. Zhang L. Reinach P. S. Tian X.. (2020). Metagenomic profiling of ocular surface microbiome changes in Meibomian gland dysfunction. Investig. Opthalmol. Vis. Sci. 61:22. doi: 10.1167/iovs.61.8.22, PMID: 32673387 PMC7425691

[ref53] Zhou Y. Holland M. J. Makalo P. Joof H. Roberts C. H. Mabey D. C.. (2014). The conjunctival microbiome in health and trachomatous disease: a case control study. Genome Med. 6:99. doi: 10.1186/s13073-014-0099-x, PMID: 25484919 PMC4256740

[ref54] Zilliox M. J. Gange W. S. Kuffel G. Mores C. R. Joyce C. De Bustros P.. (2020). Assessing the ocular surface microbiome in severe ocular surface diseases. Ocul. Surf. 18, 706–712. doi: 10.1016/j.jtos.2020.07.007, PMID: 32717380 PMC7905829

[ref55] Zou L. Ruan F. Huang M. Liang L. Huang H. Hong Z.. (2020). SARS-CoV-2 viral load in upper respiratory specimens of infected patients. N. Engl. J. Med. 382, 1177–1179. doi: 10.1056/NEJMc2001737, PMID: 32074444 PMC7121626

